# RISK FACTORS FOR EARLY POSTOPERATIVE COMPLICATIONS IN ACUTE COLITIS IN THE ERA OF BIOLOGIC THERAPY

**DOI:** 10.1590/0102-672020230052e1770

**Published:** 2023-10-23

**Authors:** Lucas Faraco Sobrado, Fernando Noboru Cabral Mori, Carolina Bortolozzo Graciolli Facanali, Mariane Gouvea Monteiro Camargo, Sérgio Carlos Nahas, Carlos Walter Sobrado

**Affiliations:** 1 Universidade de São Paulo, Coloproctology Division, Gastroenterology Department, Faculty of Medicine – São Paulo (SP), Brazil.

**Keywords:** Proctocolitis, Postoperative complications, Colitis, Colectomy, Risk factors, Biological therapy, Proctocolite, Complicações pós-operatórias, Colite, Colectomia, Fatores de risco, Terapia biológica

## Abstract

**BACKGROUND:**

Despite major advances in the clinical treatment of inflammatory bowel disease, some patients still present with acute colitis and require emergency surgery.

**AIMS:**

To evaluate the risk factors for early postoperative complications in patients undergoing surgery for acute colitis in the era of biologic therapy.

**METHODS:**

Patients with inflammatory bowel disease admitted for acute colitis who underwent total colectomy at a single tertiary hospital from 2012 to 2022 were evaluated. Postoperative complications were graded according to Clavien-Dindo classification (CDC). Patients with more severe complications (CDC≥2) were compared with those with less severe complications (CDC<2).

**RESULTS:**

A total of 46 patients underwent surgery. The indications were: failure of clinical treatment (n=34), patients’ or surgeon's preference (n=5), hemorrhage (n=3), toxic megacolon (n=2), and bowel perforation (n=2). There were eight reoperations, 60.9% of postoperative complications classified as CDC≥2, and three deaths. In univariate analyses, preoperative antibiotics use, ulcerative colitis diagnosis, lower albumin levels at admission, and preoperative hospital stay longer than seven days were associated with more severe postoperative complications.

**CONCLUSIONS:**

Emergency surgery for acute colitis was associated with a high incidence of postoperative complications. Preoperative use of antibiotics, ulcerative colitis, lower albumin levels at admission, and delaying surgery for more than seven days were associated with more severe early postoperative complications. The use of biologics was not associated with worse outcomes.

## RESUMO

**RACIONAL:**

Apesar dos enormes avanços no tratamento das doenças inflamatórias intestinais (DII), alguns pacientes apresentam quadros de colite aguda refratária ao tratamento clínico, e necessitam de cirurgia de urgência.

**OBJETIVOS:**

Avaliar os fatores de risco associados com complicações pós-operatórias precoces nos pacientes com colite aguda submetidos a colectomia na era das terapias biológicas.

**MÉTODOS:**

Pacientes com DII admitidos com colite aguda grave submetidos a colectomia total em hospital terciário no período de 2012 a 2022 foram analisados. As complicações pós-operatórias foram graduadas de acordo com a classificação Clavien-Dindo (CCD). Pacientes com complicações mais graves (CCD≥2) foram comparados com os menos graves (CCD<2).

**RESULTADOS:**

Foram submetidos a cirurgia 46 pacientes. As indicações foram: falha do tratamento conservador (n=34), preferência do paciente ou do cirurgião (n=5), hemorragia (n=3), megacólon tóxico (n=2) e perfuração intestinal (n=2). Reoperação foi necessária em oito pacientes, 60,9% tiveram complicações classificadas como CCD≥2, e três pacientes foram a óbito. Análise univariada identificou que uso de antibióticos no pré-operatório, diagnóstico de colite ulcerativa, hipoalbuminemia na admissão e período de internação maior que sete dias foi associada à complicações pós-operatória mais graves.

**CONCLUSÕES:**

Pacientes com colite aguda submetidos a cirurgia de urgência apresentaram alta taxa de complicações pós-operatórias. Uso pré-operatório de antibióticos, diagnóstico de retocolite ulcerativa, hipoalbuminemia na admissão e retardo na operação por mais que sete dias, esteve associado a complicações pós-operatórias mais graves. Uso de biológicos não se associou a piores desfechos.

## INTRODUCTION

Inflammatory bowel disease (IBD) is characterized by chronic inflammation of the bowel. In cases of ulcerative colitis (UC), the inflammation is restricted to the colon and rectum, whereas in Crohn's disease (CD), it can affect the gastrointestinal tract from the oral cavity to the anus^16^. IBD is characterized by intermittent periods of flares that can manifest with acute symptoms such as abdominal pain, fever, elevation in serum inflammatory markers, bloody diarrhea, and bowel dilation. These criteria form the definition of acute colitis. Historically, Truelove & Witts criteria are used for defining the severity of acute colitis^26^.

Since the approval by the Federal and Drug Administration (FDA) of biologic medications for CD in 1998 and for UC in 2005, the treatment for IBD has changed profoundly and a cohort of patients can delay or avoid surgery due to better clinical control of their symptoms^12^. At present, surgical treatment is recommended for patients who are refractory to maximal medical treatment; for those who develop cancer or multifocal dysplasia; or, in the acute setting, for complications such as acute severe colitis failing rescue therapy, presence of hemorrhage, bowel perforation, or toxic megacolon^9,24^. Despite major advances in the clinical treatment of these patients, acute complications are still a major indication for abdominal colectomy^4,25^.

Treatment for acute colitis includes intravenous steroids and rescue therapy with infliximab, a tumor necrosis factor-alpha inhibitor (anti-TNF), or cyclosporine. Other anti-TNF agents such as golimumab and adalimumab, although targeted for moderate and severe IBD in clinical practice, are not used for rescue therapy^13^. For refractory cases, emergency total colectomy with an end-ileostomy is the procedure of choice. Previous literature has shown that emergency surgery is associated with higher risks of postoperative complications^10,23^ and that delaying surgery after an unsuccessful clinical treatment also correlates with worse outcomes^20,22^.

To date, there is still a debate whether biologic therapy with infliximab increases postoperative complications, although most recent data suggests it does not^17^. To the authors’ best understanding, few studies have evaluated the postoperative outcomes following emergency surgery in patients who have failed anti-TNF therapy^3,6,19^. Hence, the aim of this study was to evaluate the risk factors associated with severe postoperative complications in acute colitis in the era of biologic therapies.

## METHODS

All patients with IBD admitted with acute colitis and who underwent total colectomy at a single tertiary hospital from January 2012 to February 2022 were included. A prospectively maintained database of surgical patients was accessed and patients were identified. Data were collected retrospectively in order to evaluate the risk factors associated with severe postoperative complications.

Data collected included demographics, body mass index (BMI), diagnosis, comorbidities, American Society of Anesthesiologists (ASA) score, previous treatments and hospitalizations for acute colitis, laboratory work-up, preoperative antibiotics, nutritional support, and perioperative blood transfusion. The perioperative period was defined as up to seven days before or after the surgical procedure. Inpatient treatment, including intravenous corticosteroids and rescue therapy with anti-TNF infusions or cyclosporine were recorded, along with preoperative and total hospital stay.

### Statistical analysis

Qualitative data were expressed as absolute and relative frequencies, whereas quantitative data were expressed as means, medians, standard deviations, minimum and maximum values.

The association between demographic and treatment variables with postoperative complications was evaluated using the chi-square test, Fisher's exact test, or likelihood ratio analyses. Quantitative variables were compared using the Student's *t*-test or the Mann-Whitney test. For statistical purposes, Clavien-Dindo classification were separated into two groups according to severity of complications: grades 0 and 1 (less severe) and 2, 3, 4, or 5 (more severe)^7^ (Table 1). Univariate analyses were performed to determine the odds ratio (OR) with a 95% confidence interval (CI). Multivariate analyses could not be performed due to the limited number of events.

**Table 1:** Clavien-Dindo Classification^7^.

Grade	Definition
0	No complication
1	Any deviation from the normal postoperative course. Allowed physical therapy and medications such as: analgesics, anti-emetics, antipyretics, electrolytes and diuretics
2	Requiring pharmacological treatment with drugs other than such allowed for grade I complications, including total parenteral nutrition
3	Requiring surgical, endoscopic or radiological intervention
4	Life-threatening complication requiring intermediate or intensive care management
5	Death

Analyses were performed with the Statistical Package for Social Sciences (SPSS) — IBM software for Windows, version 22.0, and a p-value (p) equal to 0.05 or lower was considered significant.

## RESULTS

A total of 46 patients underwent surgical treatment for acute colitis. Of these, 30 patients had been previously treated with biologic therapy and 19 were receiving biologics at the time of hospital admission.

In regard to in-hospital clinical treatment, 34 patients received intravenous steroids and six received rescue therapy with infliximab at 5 mg/kg. The reasons for not receiving in-hospital biologic therapy included previous failure of multiple biologics or concurrent use at admission (n=28), contraindication or previous adverse reactions (n=4), hemodynamic instability (n=3), toxic megacolon (n=2), large bowel stricture on imaging (n=2), and recent tuberculosis treatment (n=1). One patient was diagnosed with *Clostridioides difficile* and another with cytomegalovirus (CMV) colitis but both failed clinical treatment and underwent surgical treatment. No patients received cyclosporine. The patient's characteristics and demographics are summarized in Table 2.

**Table 2:** Demographic and clinical data.

Variable		Total cohort (n=46)
Age at surgery (years)		41.3±16.3
Gender		
	Male	21 (45.7)
	Female	25 (54.3)
BMI (kg/m²)		21.2±4.1
IBD diagnosis		
	UC	25 (54.3)
	CD	17 (37)
	Indeterminate colitis	4 (8.7)
Time since diagnosis (months)		88.3 (0–424.8)
Previous hospitalizations due to acute colitis		31 (67.4)
Active smoker		12 (26.1)
ASA score		
	II	38 (82.6)
	III	7 (15.2)
	IV	1 (2.2)
Clinical comorbidities		18 (39.1)
Previous use of biologic therapy		
	No	16 (34.8)
	One medication	12 (26.1)
	Two or more	18 (39.1)
Laboratory work-up at hospital admission		
	Hemoglobin level (g/dL)	9.5±2.5
	CRP level (mg/L)	68.5 (1.6–345.9)
	Albumin level (g/dL)	2.93±0.74
Current use of biologics at hospital admission		19 (41.3)
Received intravenous steroids		34 (73.9)
Received rescue therapy with anti-TNF		6 (14)
Preoperative antibiotics use		32 (69.6)
Laboratory work-up prior to surgery		
	Hemoglobin level (g/dL)	9.8±1.7
	CRP level (mg/dL)	37.6 (0.3–306.5)
Perioperative blood transfusion		26 (56.5)
Preoperative nutritional support		23 (50)
Preoperative hospital stay (days)		8 (1–57)
Surgical approach		
	Open or converted	25 (54.3)
	Laparoscopic	21 (45.7)
Postoperative hospital stay (days)		12.5 (4–66)
Total hospital stay (days)		29 (5–82)
Follow-up since surgery (months)		21.5 (0.7–116.9)

Data are presented as n (%), mean±standard deviation, or median (range). BMI: body mass index; IBD: inflammatory bowel disease; UC: ulcerative colitis; CD: Crohn's disease; ASA: American Society of Anesthesiologist; CRP: C-reactive protein.

The surgical indications were the failure of clinical treatment (n=34), patient's or surgeon's preference (n=5), hemorrhage (n=3), toxic megacolon (n=2), and bowel perforation (n=2). In all patients, the rectum was divided with a linear stapler and left inside the abdominal cavity, followed by distal bowel washout, and positioning of a pelvic drain next to the rectal stump, which is the standard practice at the authors’ institution.

Postoperative complications are shown in Table 3. There were a total of 59 postoperative complications and 15 multiple complications, which are listed in Table 4. A total of eight patients (17.4%) had to undergo reoperation due to evisceration (n=2), stoma retraction (n=1), ischemia (n=1), intraabdominal abscess (n=1), rectal stump dehiscence (n=1), small bowel perforation (n=1), and intraabdominal hemorrhage following anticoagulation for deep vein thrombosis (n=1). Three patients (6.5%) died during hospitalization.

**Table 3:** Postoperative complications classified according to Clavien-Dindo^7^.

Grade	n (%)
0	15 (32.6)
1	3 (6.5)
2	16 (34.8)
3	4 (8.7)
4	5 (10.9)
5	3 (6.5)

**Table 4:** Postoperative complications.

Complication	n
Ileus	12
Required total parenteral nutrition	11
High-output ileostomy	4
Bloodstream infections	3
Death	3
Rectal stump dehiscence	3
Superficial surgical site infection	2
Organ-space surgical site infection	2
Evisceration	2
Pain requiring patient-controlled analgesia	2
Acute kidney injury	2
Ileostomy retraction	1
Ileostomy ischemia	1
Deep vein thrombosis	1
Portal vein thrombosis	1
Small bowel perforation	1
Bleeding esophageal varices	1
Surgical site hemorrhage	1
Iatrogenic pneumothorax	1
Parastomal hernia	1
Bleeding due to duodenal ulcer	1
Gangrenous pyoderma	1
Lower urinary tract infection	1
Pyelonephritis	1
Total	59

The preoperative antibiotic use (p=0.003; p<0.05), UC diagnosis (p=0.007; p<0.05), lower albumin levels at admission (p=0.035; p<0.05), and preoperative hospital stay longer than seven days (p=0.040; p<0.05) were associated with more severe early postoperative complications (CDC≥2) in univariate analyses. Perioperative blood transfusion (p=0.053; p>0.05), comorbidities (p=0.060; p>0.05), and no prior admissions to acute colitis (p=0.064; p>0.05) approached but did not meet significance. Other factors such as sex, BMI, American Society of Anesthesiologists (ASA) score, previous or current biologic therapy, hemoglobin and C-reactive protein (CRP) at admission or at the time of surgery, in-hospital steroids, preoperative nutritional support, and surgical approach were not associated with more severe complications (Table 5).

**Table 5:** Comparison between patients with more and less severe postoperative complications following colectomy.

Variable		Clavien-Dindo		OR	95%CI		p
		0 and 1	2, 3, 4 and 5		Inferior	Superior	
Age (years)		36.6±13	44.4±17.7	1.032	0.992	1.073	0.115*
Sex							
	Male	8 (38.1)	13 (61.9)	1.00			0.895
	Female	10 (40)	15 (60)	0.92	0.28	3.03	
	BMI (kg/m²)	21.1±4.3	21.3±4.1	1.008	0.872	1.165	0.916*
Diagnosis							
	UC	7 (28.0)	18 (72.0)	1.00			0.007†
	CD	11 (64.7)	6 (35.3)	0.21	0.06	0.80	
	Indeterminate colitis	0 (0)	4 (100)	‡			
	Time since diagnosis (months)	78.1 (2.8–315.2)	102.1 (0–424.8)	1.005	0.997	1.013	0.155§
Previous admissions due to acute colitis							
	No	3 (20.0)	12 (80.0)	1.00			0.064
	Yes	15 (48.4)	16 (51.6)	0.27	0.06	1.14	
Smoking							
	No	16 (47.1)	18 (52.9)	1.00			0.090//
	Yes	2 (16.7)	10 (83.3)	4.44	0.84	23.39	
ASA score							
	II	16 (42.1)	22 (57.9)	1.00			
	III	2 (28.6)	5 (71.4)	1.82	0.31	10.59	0.478†
	IV	0 (0)	1 (100)	‡			
Clinical comorbidities							
	No	14 (50.0)	14 (50.0)	1.00			0.060
	Yes	4 (22.2)	14 (77.8)	3.50	0.92	13.31	
Previous use of biologics							
	No	4 (25.0)	12 (75.0)	1.00			0.152
	Yes	14 (46.7)	16 (53.3)	0.38	0.10	1.46	
Current use of anti-TNF at admission							
	No	8 (29.6)	19 (70.4)	1.00			0.116
	Yes	10 (52.6)	9 (47.4)	0.38	0.11	1.29	
Laboratory work-up at admission							
	Hemoglobin (g/dL)	10.1±2.0	9.2±2.7	0.855	0.662	1.104	0.233*
	CRP (mg/dL)	61.0 (2.9–212.3)	74.7 (1.6–345.9)	1.004	0.995	1.013	0.445£
	Albumin (g/dL)	3.21±0.62	2.75±0.76	0.379	0.148	0.970	0.035*
In-hospital steroids							
	No	6 (50)	6 (50)	1.00			0.495//
	Yes	12 (35.3)	22 (64.7)	1.83	0.48	6.95	
Preoperative antibiotics							
	No	10 (71.4)	4 (28.6)	1.00			0.003
	Yes	8 (25.0)	24 (75.0)	7.50	1.83	30.68	
Laboratory work-up at surgery							
	Hemoglobin (g/dL)	10.2±1.4	9,5±1,8	0.775	0.532	1.128	0.184*
	CRP (mg/L)	38.5 (2.4–74.7)	28.6 (0.3–306.5)	1.009	0.995	1.024	0.673§
Perioperative blood transfusion							
	No	11 (55.0)	9 (45.0)	1.00			0.053
	Yes	7 (26.9)	19 (73.1)	3.32	0.96	11.42	
Preoperative nutritional support							
	No	11 (47.8)	12 (52.2)	1.00			0.227
	Yes	7 (30.4)	16 (69.6)	2.10	0.63	7.01	
Days in hospital until surgery							
	≤7 days	12 (54.5)	10 (45.5)	1.00			0.040
	>7 days	6 (25.0)	18 (75.0)	3.60	1.03	12.54	
Surgical approach							
	Open or converted	8 (32.0)	17 (68.0)	1.00			0.280
	Laparoscopic	10 (47.6)	11 (52.4)	0.52	0.16	1.72	
Postoperative in-hospital period (days)		7.0 (4–11)	23.5 (6–66)	1.768	1.195	2.615	<0.001§
Total in-hospital period (days)		12.0 (5–58)	37.5 (16–82)	1.094	1.034	1.158	<0.001§

* student's *t*-test;

† likelihood ratio test;

‡ not estimated;

§ Mann-Whitney's test;

// Fisher's exact test.

CI: confidence interval; p: chi-square test; BMI: body mass index; UC: ulcerative colitis; CD: Crohn's disease; ASA: American Society of Anesthesiologist; TNF: tumor necrosis factor; CRP: C-reactive protein.

The histopathological examination of the specimen revealed colitis (n=44) and incidental findings of cancer (n=2), staged as pT1N0 and pT2N0. Regarding these two patients, one had previous high-grade dysplasia under surveillance and the other had primary sclerosing cholangitis as risk factors for neoplasia. Both had strictures on preoperative imaging.

## DISCUSSION

The main findings of this study were that postoperative complications following surgery for acute colitis are common, and the risk factors associated with more severe complications are UC diagnosis, use of preoperative antibiotics, lower albumin levels at admission, and preoperative hospital stay greater than seven days.

Previous studies have evaluated the surgical outcomes for acute colitis^6,13,14,19,20,22^, but mostly in patients who had never been exposed to biologics, which may not necessarily translate to current practice. In this manuscript, neither in-hospital steroids nor previous or current use of biologics at admission were associated with more severe postoperative complications. There is an ongoing debate about the use of anti-TNF and surgical results, although most recent data suggest it does not influence postoperative outcomes^27^. Other authors have also shown that rescue therapy for severe colitis is not associated with an increased risk of postoperative complications^18,19^. It has been suggested that third-line therapy could be used if conventional therapy fails, and a systematic review on this subject reported adverse events in 23% of patients and a colectomy rate of 42.3% at 12 months^17^. From a surgeon's perspective, this is concerning because the addition of another immunosuppressant and a delay to surgery could have negative impacts on postoperative outcomes. Large bowel preservation might not be the ultimate goal for all patients since some have long-standing disease, have failed multiple previous medications and may have worsening quality of life. Of patients admitted for acute colitis and treated successfully with medications, almost half require colectomy during follow-up^1,2^.

It is well known that infliximab is safe and effective for patients with acute severe colitis^11^. However, not all patients are candidates for clinical treatment. In this series, reasons for not receiving biologics included previous severe adverse reactions, hemodynamic instability, toxic megacolon, large bowel stricture on imaging, and recent tuberculosis treatment.

In the past decades, there has been a surge in many new medical therapies for IBD. This has made the optimal timing of surgery less clear. It depends largely on clinical expertise and shared decision-making between the multi-disciplinary team and the individual patient. In the acute setting, a delay to surgery has been associated with an increased risk for postoperative complications^20,22^, a finding confirmed by our study, considering a delay of seven days.

Current guidelines recommend the administration of intravenous steroids at admission and if no improvement is seen after three days, rescue therapy with biologics should be considered and the surgical team consulted. In non-responders, total colectomy with an end-ileostomy is the procedure of choice and should not be delayed beyond seven days of clinical treatment^8^. The operative approach, whether open or laparoscopic, did not influence postoperative outcomes in this study, which is consistent with other series^3^.

In the present study, 67.4% of patients experienced at least one postoperative complication and there were three deaths. This possibly reflects the severity of the disease and the adverse risk profile of these patients; 17.4% were ASA III or IV and 39.1% had other comorbidities besides IBD. Patients with severe complications were more likely to have prolonged hospital stay. Powar et al., in a similar study, reported severe complications in nearly one-third of patients^19^.

Patients admitted with acute colitis commonly have systemic signs of inflammation. This can be reflected by anemia, low albumin, and elevated CRP. The present study shows that only a low albumin level at admission was associated with worse outcomes. Interestingly, preoperative nutritional support had no influence on the outcomes. Perioperative blood transfusion also did not meet significance (p=0.053). Other studies have associated preoperative blood transfusion with an increased risk for both infectious and non-infectious postoperative complications in CD^15^. However, in patients with hemorrhage or hemodynamic instability with severe anemia, blood transfusion should not be delayed.

The benefits of antibiotic use in acute colitis are uncertain and not routinely supported by the literature^5^. Nevertheless, patients with severe colitis are often treated with third-generation cephalosporin and metronidazole due to concerns about bacterial translocation and sepsis. Our data show that antibiotic use was associated with more severe postoperative complications. A potential explanation for this is the selective use of antibiotics in more severe cases. There is also ample evidence of dysregulation of the gut microbiome in IBD, accounting also for its pathogenesis. Misuse of antibiotics not only alters the gut microbiome but also increases the risk of developing antimicrobial-resistant strains. As mentioned, the prescription of antibiotics is not systematic in the literature in cases of acute colitis^21^. Larger studies are needed to better define the role of antibiotic therapy in this setting.

It is unclear why UC was associated with more severe postoperative complications than CD. Perhaps, the need of clinicians and patients to exhaust all clinical treatments for UC, in an effort to avoid colectomy, delays surgical treatment, whereas in CD, it is possible that the surgeon and the gastroenterologist are more inclined to refer for surgery earlier. A history of prior admissions was also associated with less severe complications, which may be due to more prompt surgical consultation and sooner surgery.

This study has multiple strengths and limitations. First, due to its relatively small sample size, multivariate analyses could not be performed, which limits the interpretation of the variables as independent factors. Second, its retrospective design does not allow for causality to be established and other factors may play a role in explaining the differences observed. Third, patients could not be classified according to Truelove & Witts criteria because not all clinical data were available. In terms of strengths, it is a single institution cohort, which allows for a high consistency in terms of clinical and surgical treatment for severe colitis. To the authors’ knowledge, no previous research has investigated risk factors for early postoperative complications in acute colitis in Brazil. Finally, this study provides important data on early postoperative outcomes in the era of biologics for patients with acute colitis and reflects current practice in IBD management.

## CONCLUSIONS

Emergency surgery for acute colitis is associated with a high incidence of postoperative complications. The risk factors identified in this study that were associated with the more severe early postoperative complications included preoperative use of antibiotics, diagnosis of ulcerative colitis, lower albumin levels at admission, and a delay in surgery greater than seven days. An association between the use of biologics and negative outcomes was not proven.
